# Apexification Management of Mandibular Second Premolar with a Blunderbuss Apex and Periapical Lesion of an Adult Patient

**DOI:** 10.1155/2019/7546842

**Published:** 2019-04-09

**Authors:** Chenshuang Li, Zhong Zheng, Xiaohong Deng, Lan Zhang, Baoyan Wang, Qingyu Guo, Min Zou

**Affiliations:** ^1^College of Stomatology, Xi'an Jiaotong University, Xi'an, Shaanxi 710004, China; ^2^Division of Growth and Development, Section of Orthodontics, School of Dentistry, University of California, Los Angeles, Los Angeles, CA 90095, USA; ^3^Department of Endodontics, College of Stomatology, Xi'an Jiaotong University, Xi'an, Shaanxi 710004, China; ^4^Department of Oral Mucosal Diseases, College of Stomatology, Xi'an Jiaotong University, Xi'an, Shaanxi 710004, China; ^5^Department of Pediatric Dentistry, College of Stomatology, Xi'an Jiaotong University, Xi'an, Shaanxi 710004, China; ^6^Department of Orthodontics, College of Stomatology, Xi'an Jiaotong University, Xi'an, Shaanxi 710004, China

## Abstract

Apexification is widely applied in teenager patients but rarely used in the adult population. Instead, artificial apical barrier with mineral trioxide aggregate (MTA) is clinically accepted, and spontaneous apical closure in nonvital immature teeth of adult patients has rarely been encountered while only apexification of the maxillary incisors in adult patients has been reported. The aim of this case report is to share a successful apexification application in the mandibular premolar with a blunderbuss apex and periapical lesion of an adult patient by using calcium hydroxide: radiographically, spontaneous hard tissue barrier has been established, and narrowing canal space and decreasing area of periapical shadow were documented without complications. Taken together, our study indicates that patient's age and tooth position may not be the critical limitation for apexification.

## 1. Introduction

Dens evaginatus (DE) is a dental development anomaly that can arise in any teeth. Fracture and abrasion of the DE may cause pulp infection and necrosis, while in adolescents, fracture and abrasion of the DE can also lead to immature tooth root development. Meanwhile, an immature apex always holds a blunderbuss shape that fails to limit the compaction material in the canal, which makes it difficult to fill the canal efficiently during root canal therapy [1]. Recently, some studies have shown that mineral trioxide aggregate (MTA) can be used in one-visit apexification treatments for teeth with large canals and an open apex in adults [1]. However, for a long time, information about the definite clinical effect of apexification of open apex teeth of adult patients by using calcium hydroxide [Ca(OH)_2_] has been lacking. In 2013, Costa et al. [2] reported a successful application of apexification at the maxillary lateral incisor of an adult patient, while in 2015, Caliskan and Kaval [3] published a manuscript that documented Ca(OH)_2_ treatment that effectively established spontaneous hard tissue barrier in the upper central incisors of three adult patients. These are the only currently available studies that showed the efficacy of apexification in adults, which are limited in the maxillary incisors. Here, we reported a case that successfully used Ca(OH)_2_ treatment to induce apex closure in a second premolar of an adult patient with long-term chronic apical periodontitis.

## 2. Case Report

A 24-year-old male complained of a sinus tract located in the buccal gingiva of the mandibular left posterior area for 12 years. The patient experienced spontaneous toothaches in his left posterior region of the mandible which vanished when antibiotics and anti-inflammatory agents were taken 12 years ago. Subsequently, a sinus tract was found in the buccal gingiva of the patient's mandibular left posterior area. Although the patient felt uncomfortable while eating, he did not have it checked by any dentist until this hospital visit.

**Figure 1:**
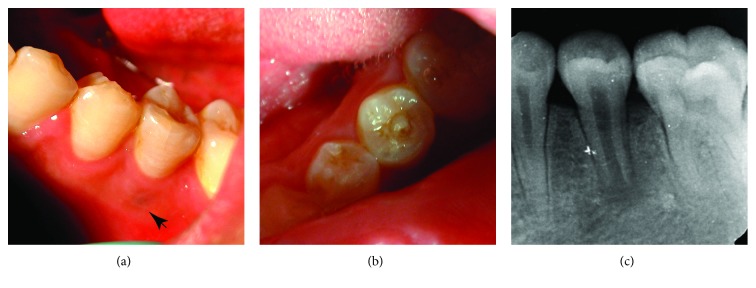
Clinical photograph and periapical radiograph of this case. (a) The clinical photograph shows a violet-blue patch with a diameter of 2 mm in the buccal gingiva of tooth #20. (b) The clinical photograph shows an attrited abnormal center cusp at the center of the occlusal surface of tooth #20. (c) The periapical radiograph shows a radiolucent lesion at the periapical area of tooth #20 with a wide open apex.

Intraoral examination revealed a violet-blue patch in the buccal gingiva of tooth #20 with a diameter of 2 mm. At the center of the patch, a closed sinus tract was noticed (Figure 1(a)). Abraded dens evaginatus was found in the center of the occlusal surface of tooth #20, and a fine explorer could not be inserted into the center of the fractured dens evaginatus (Figure 1(b)). The tooth showed a negative response to the cold test with Endo Ice and hot test with heated base plate gutta-percha, sensitivity to percussion, and no mobility, whereas there was no significant periodontal pocket around. An immature root with a blunderbuss apex and a periapical shadow with the size about 4 mm × 3 mm were demonstrated by X-ray radiography (Figure 1(c)). Thus, the clinical diagnosis of tooth #20 was pulp necrosis with chronic periapical periodontitis.

**Figure 2:**
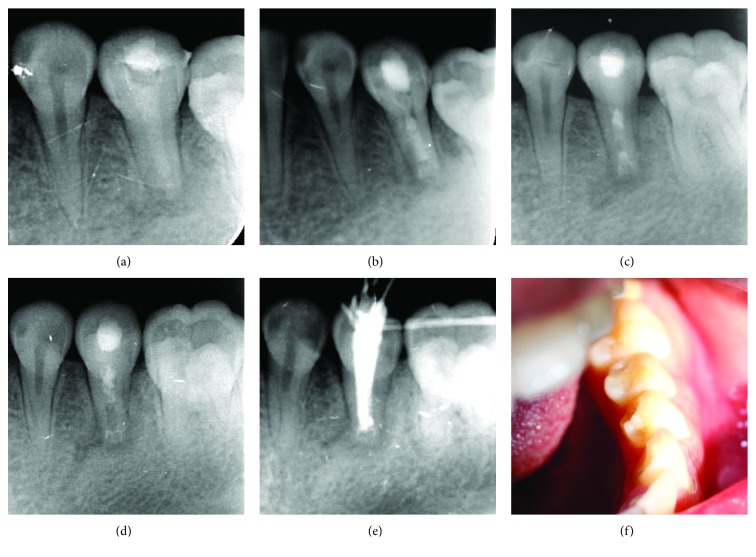
Follow-up periapical radiographs of this case. (a) The radiograph made directly after the initial treatment shows a compact filling of Ca(OH)_2_ paste with working length, an open apex, and a radiolucent lesion of tooth #20. (b) The radiograph made 3 months after the initial treatment shows absorption of Ca(OH)_2_ paste in the canal and a slightly narrowed root apex. (c) The radiograph made after a new Ca(OH)_2_ paste was placed. (d) 7 months after the initial treatment shows healing of the periapical bone, more reduction of the root canal space, and a significant root-end barrier. (e) The radiograph made after the root canal was filled with gutta-percha shows a significant root-end barrier and a compact filling. (f) The clinical photograph taken 2 years after the treatment was finished shows a sound tooth #20 without discoloration and disappearance of the patch in the buccal gingiva of tooth #20.

Without anesthesia, the tooth was accessed. Accompanied by a copious hemorrhage, the patient experienced mild pain upon reaching the apex area with a barbed broach. The pulp chamber was abundantly irrigated with 3% hydrogen peroxide and 0.9% saline until no significant hemorrhagic secretion was noticed. As the tooth had a blunderbuss apex, accurate root canal length cannot be measured by electronic root canal length measurement devices; thus, the length of the canal was measured with an X-ray by placing a #40 gutta-percha in the canal and measuring the length of the gutta-percha. The canal was carefully dried, and a little cotton pellet was put into the canal as drainage. One week later, the patient reported no symptoms since the first appointment. There was no hemorrhage upon reentry, and the Ca(OH)_2_ paste mixed with silicone oil was placed into the root canal. Glass-ionomer cement was used to seal the access (Figure 2(a)). Three months after the first Ca(OH)_2_ treatment, the patient remained asymptomatic, and the patch disappeared. The X-ray examination showed Ca(OH)_2_ absorption in the canal (Figure 2(b)). After removing the Ca(OH)_2_ paste by 0.9% saline, a thin hard barrier was detected, while an open apex was still detectable by X-ray in the treated tooth (Figure 2(c)). Therefore, a second injection of Ca(OH)_2_ paste was applied and the access was closed with glass-ionomer cement similar to the initial treatment. Seven months after the first Ca(OH)_2_ treatment (four months after the second treatment), periapical radiography demonstrated the absorption of Ca(OH)_2_ paste, significantly narrowed root canal, obviously established root-end barrier, and progressively healed periapical bone with minor radiolucency around the apex (Figure 2(d)). An apical probing with a #40 K-file was used to confirm the apical barrier, and there was no exudate drainage evident. Then, the canal was washed, dried, and filled with gutta-percha (Figure 2(e)). The tooth was rebased with glass-ionomer cement and sealed with composite resin.

An intraoral examination done two years after the composite resin sealing showed tooth #20 without discoloration and disappearance of the patch in the buccal gingiva (Figure 2(f)). The patient did not return for subsequent follow-ups.

## 3. Discussion

As a classical apexification medication, Ca(OH)_2_ paste can not only control the infection but also induce the root continued development. Indeed, Ca(OH)_2_ paste has been shown to be successful 100% of the time in adolescents in several studies [4, 5]. It is worth noting that almost all cases with successful apexification are reported in the adolescent population [6–8]. Here, by demonstrating the efficacy of Ca(OH)_2_ treatment in apex closure of an adult patient suffering from a long-term chronic apical periodontitis, our current case report echoed the previous publication that described the successful establishment of spontaneous hard tissue barrier in the upper central incisors in adults by Ca(OH)_2_ application [3]. And thus, our current report supports the challenging of the paradigm that apexification only mainly works on adolescents [4, 9–12] and strongly suggests that apexification can also be applied to adults at different tooth positions which is not limited to maxillary anterior teeth [2, 3].

An alternative treatment to Ca(OH)_2_ apexification is using an artificial apical barrier with MTA that allows for immediate obturation of the canal. This procedure has steadily gained popularity among clinicians because of its short treatment time. Since clinical success and apical barrier formation were used to assess the efficacy of the treatment provided with Ca(OH)_2_ and MTA, there was no statistical significance among the two drugs [13]. In addition, thin dentinal walls still present a clinical problem for MTA treatment. Erdem and Sepet [10] suggested that MTA failure in complete healing are due to the unusual width and shape of the canal, difficulties in disinfecting the canal and dentinal tubules, and the porous structure of the apical calcified barrier. Therefore, MTA cannot replace apexification using Ca(OH)_2_ because of the lack of long-term efficacy, expensive procedural costs, an unrealistic requirement of technology and facilities, and unknown undifferentiated short-term effects [7, 14]. Many other materials have also been used for apexification, but none has truly replaced Ca(OH)_2_ due to the lack of clinical success [4]. There are also reports about the use of revascularization [4], stem cell regeneration [15], and even irrigation with copious amounts of 2.5% NaOCl [14], but cases are limited and lack long-term evidence.

In summary, our current case report further demonstrated that Ca(OH)_2_ can lead to apex closure even if the tooth had a long-term chronic apical periodontitis [3], supporting the hypothesis that apexification can also be applied to adults and not limited to maxillary anterior teeth. Systematic study with more samples and different tooth positions is needed to further confirm the efficiency of apexification with Ca(OH)_2_ in adult patients.
